# An Index of Differential Diagnosis of Main Symptoms

**Published:** 1913-03

**Authors:** 


**An Index of Differential Diagnosis of Main Symptoms.** By Various Writers. Edited by HERBERT FRENCH, M.A., M.D. Oxon., F.R.C.P. Pp. xii, 1011. Bristol: John Wright & Sons Ltd. 1912.

It is impossible to indicate in a short review the whole contents of this work. The aim of the editor and his collaborators has been to help the practitioner in the diagnosis of his cases by giving full information as to the conditions under which a particular symptom may occur, and by the aid of the associated signs present, thus enable him to distinguish the disease with which he is dealing. The book is therefore arranged in encyclopædic form, the main headings being those of symptoms, and these are supplemented by a general index which gathers them together under diseases. The book is therefore meant to be used with a free reference to the general index, and on the adequacy of the latter its usefulness must largely depend. So far as we have been able to ascertain the index meets the conditions, and has evidently been compiled with great care and labour. It occupies over 150 pages of closely printed type in three columns to each page. No doubt a work of this kind will be found useful to many men to refer to for the meaning of unusual or obscure symptoms; and if employed as an addition to the more systematic works on medicine will form a useful supplement to them. Knowledge gained in this way is, however, apt to be scrappy, and if relied upon solely, to give a restricted view of disease. After all, each man works in his own way, and arrives at good results by very different means. So far as the work itself is concerned, we can recommend it as to being thoroughly well done, well illustrated, and omitting very little if anything in its extensive survey.

